# Supplementary material for: Generative design of functional organic molecules for terahertz radiation detection
Source: Digit Discov. 2025 Aug 22;4(10):2852–63. doi: 10.1039/d5dd00106d (PMC13579655; doi:10.1039/d5dd00106d)
Supplement: Supplementary file 1 [file DD-004-D5DD00106D-s001.pdf]

Supplementary Information (SI) for Digital Discovery.
This journal is © The Royal Society of Chemistry 2025

Supporting Information for:

'Generative design of functional organic molecules for terahertz radiation detection'

Zsuzsanna Koczor-Benda,[a*] Shayantan Chaudhuri,[a,b] Joe Gilkes,[a,c] Francesco Bartucca,[a] Liming Li,[a] and Reinhard J. Maurer[a,d*]

[a] Department of Chemistry, University of Warwick, Coventry, CV4 7AL, UK

[b] School of Chemistry, University of Nottingham, Nottingham, NG7 2RD, UK

[c] Centre for Doctoral Training in Modelling of Heterogeneous Systems, University of Warwick, Coventry, CV4 7AL, UK.

[d] Department of Physics, University of Warwick, Coventry, CV4 7AL, UK

E-mail: zsuzsanna.koczor-benda@warwick.ac.uk, r.maurer@warwick.ac.uk

Contents

S1 Training Database Information 2

S2 P Value Definition 2

S3 Hyperparameter Optimization for PaiNN and MACE 3

S4 Analysis of Generated Molecules 5

S5 Accuracy of P Value Predictions 5

S6 Retraining the PaiNN Property Predictor 6

S7 Analysis of PaiNN Predictions for Generated Molecules 8

S8 Chemical Space Mapping of Generated Molecules 10

S9 Retrosynthetic Paths and Vibrational Spectra of Candidate Molecules 11

S10 Structural Validation of Generated Molecules 22

S1 Training Database Information

Table SI details the sizes of the databases for each biasing iteration, before and after filtering subject to constraints relating to connectivity (a path should exist between any two atoms over bonds), uniqueness (no two structures should possess the same SMILES[1] representation), and a sanity check based on RDKit[2] to check atomic valencies. Molecules were also filtered to ensure the presence of only one thiol group, which would act as the linker to the gold nanoantenna in a terahertz radiation detector.

The iterative training procedure employed in this work slightly differs from the methodology outlined by Westermayr *et al.*[3] They performed iterative biasing by using a previously trained G-SchNet model and retraining it only on the small subset of molecules. Herein, we retrain G-SchNet from scratch in each iteration with the modified training dataset.

Iteration	Number of Generated Molecules	Number of Filtered Molecules	Number of Filtered Monothiols
0	62 592	38 688	25 890
1	84 592	56 687	41 285
2	90 238	59 110	46 028
3	92 712	58 343	45 986
4	92 918	57 339	45 043
5	93 426	55 942	46 426
6	95 702	56 358	46 799

Table SI. Sizes of databases of generated molecules for each biasing iteration, before and after filtering subject to constraints relating to connectivity, uniqueness, atomic valencies, and an additional filter for monothiolated molecules.

Table SII details the sizes of the training databases used to train G-SchNet models within each biasing iteration.

Iteration	Database Size
0	30 000
1	31 146
2	35 990
3	41 236
4	45 939
5	50 405
6	54.739

Table SII. Sizes (number of molecules) of the training databases for each biasing iteration.

S2 P Value Definition

Koczor-Benda *et al.*[4] defined the target property P to the logarithm of the orientation-averaged upconversion intensity (I_m^c) summed for the 1–30 THz frequency window:

$$\mathrm{P} = \log\left(\sum_{m\in M} \langle I_m^c \rangle\right), \tag{1}$$

where M is the set of vibrational normal modes of the molecule in the 1–30 THz (30–1000 cm^{-1}) frequency

Features	32	64	128	256	612	1024
Interactions	3	4	5	6	7	8

Table SIII. Combination of hyperparameter values tested for number of features and interactions for the PaiNN model.

range. I_m^c is defined as[4]:

$$I_m^c = C\frac{(\overline{\nu}^{\mathrm{aS}} + \overline{\nu}_m)^4}{\overline{\nu}_m}\left\langle \left|\underline{e}\,\underline{\mu}'_m\right|^2 \left|\underline{e}\,\underline{\underline{\alpha}}'_m\,\underline{e}\right|^2 \right\rangle \tag{2}$$

where C is a constant scaling factor (6.026324×10^{-42}), $\overline{\nu}^{\mathrm{aS}}$ and $\overline{\nu}_m$ are the wavenumbers of the visible laser and the normal mode, respectively, $\underline{\mu}'_m$ is the dipole derivative vector, and $\underline{\underline{\alpha}}'_m$ is the polarizability derivative tensor. The aligned terahertz and Raman in-out field polarization vectors are all denoted by $\underline{e}$. The visible laser wavelength was set to 785 nm and the temperature to 298.15 K, similarly to Koczor-Benda *et al.* [4],[5]. The scaling factor and the analytical formula for calculating the matrix elements in Equation 2 are detailed in Koczor-Benda *et al.* [4].

S3 Hyperparameter Optimization for PaiNN and MACE

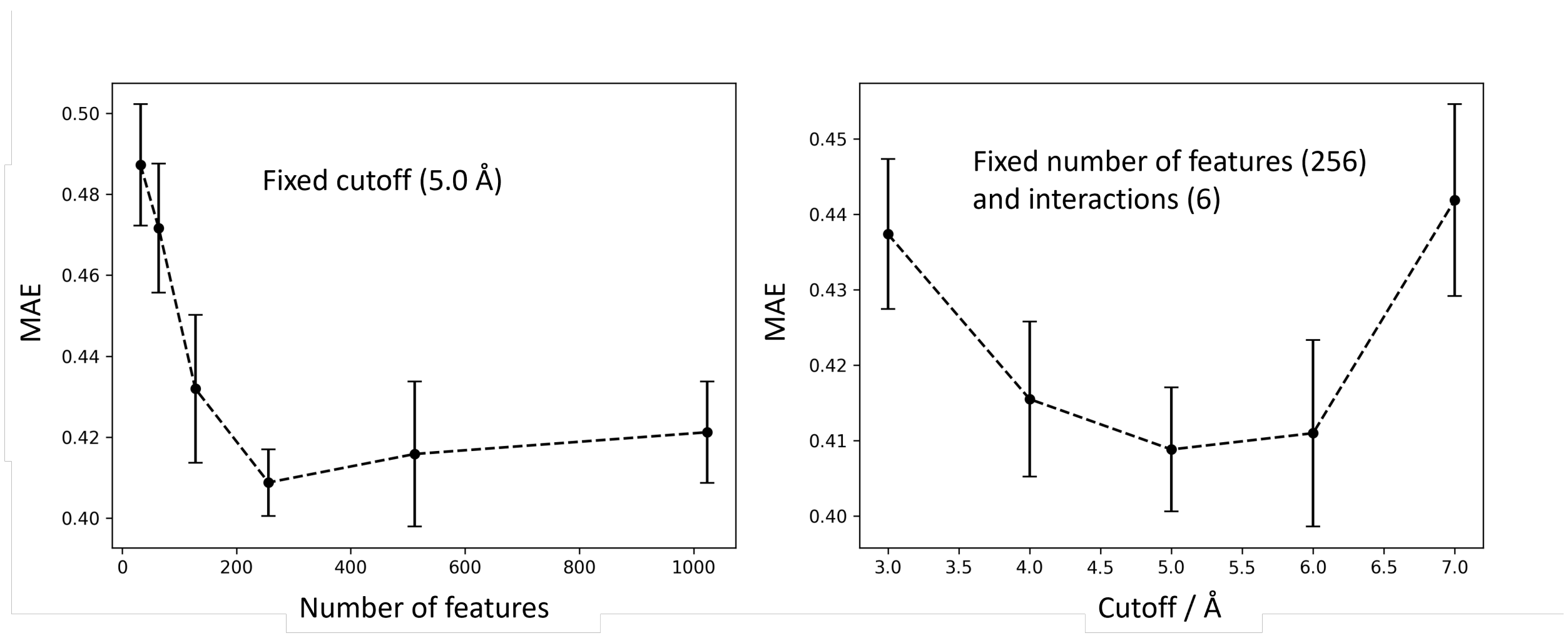

Figure S1. Hyperparameter optimization for PaiNN. For number of features, only combinations with number of interaction values specified in Table SIII were tested. The mean and standard deviation of 5-fold cross-validation results are shown for each point.

Hyperparameters	Best value
Learning Rate(lr)	0.005
Hidden Irreps(hidden_irreps)	128x0e+128x1o+128x2e
Number of Interaction Layers(num_interactions)	2
Correlation Order(correlation)	3
Angular Resolution(max_L)	2

Table SIV. The optimized values of 5 hyperparameters for MACE.

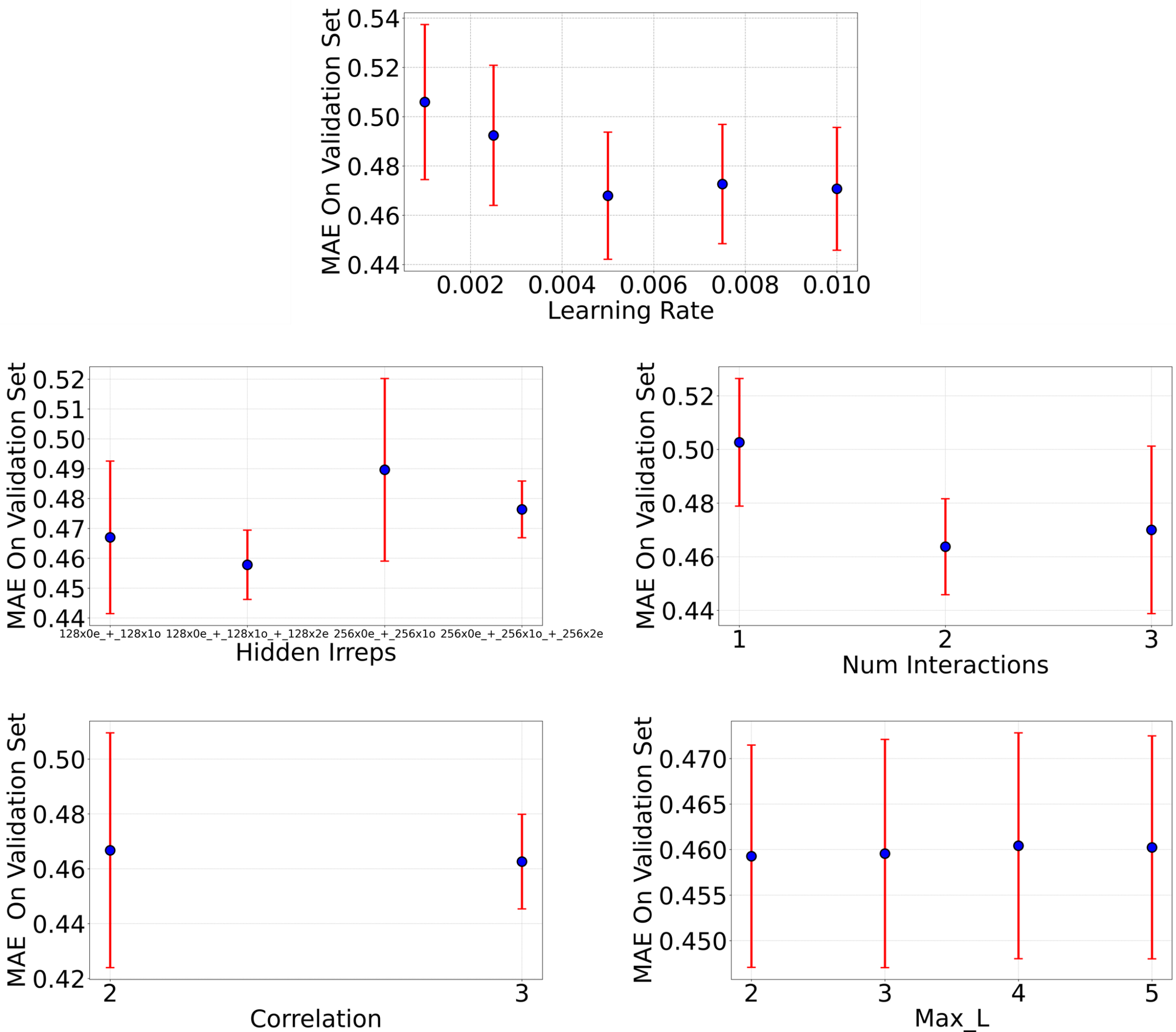

Figure S2. Hyperparameter optimization for MACE with 5-fold cross-validation.

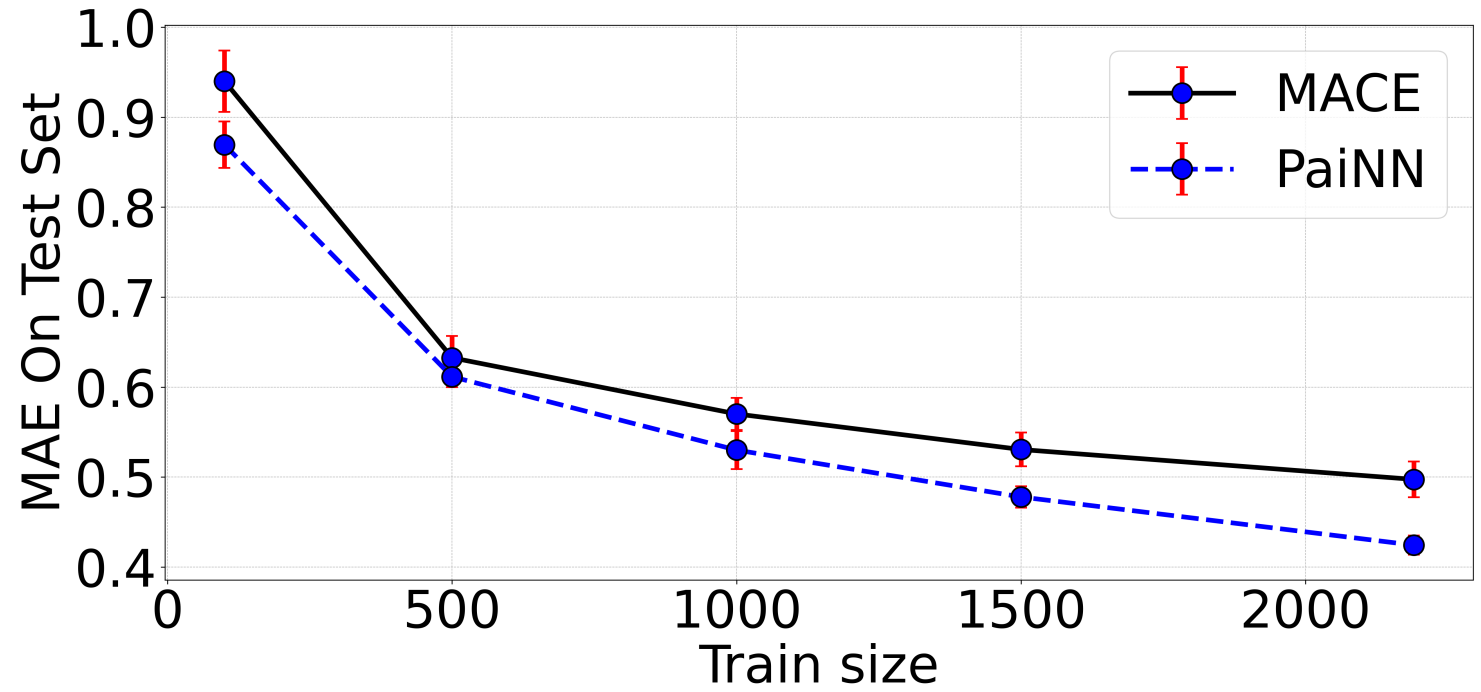

Figure S3. Mean absolute error (MAE) of MACE and PaiNN predictions for P values on the test set of the THz database as a function of training set size, using 5-fold cross-validation.

S4 Analysis of Generated Molecules

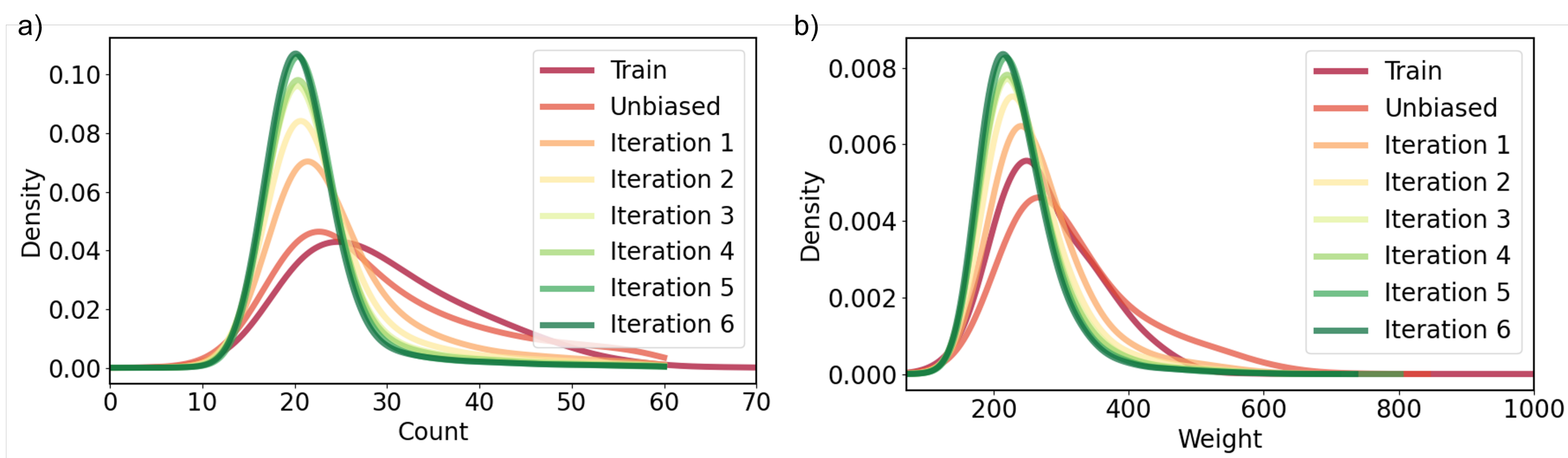

Figure S4. a) Number of atoms and b) molecular weight distribution of training and generated molecules.

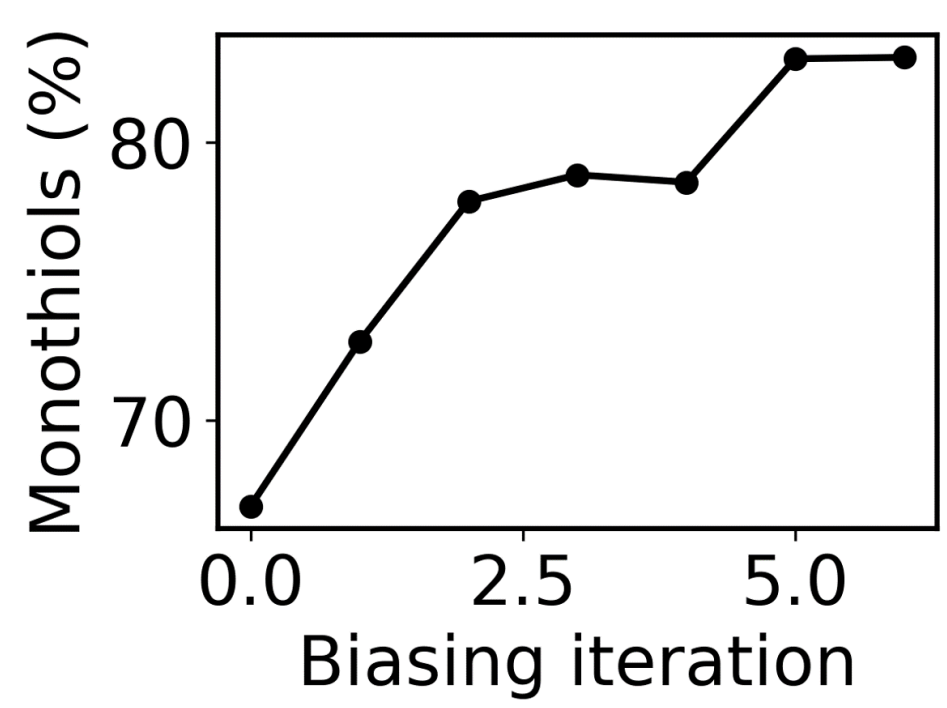

Figure S5. Proportion of molecules containing a single thiol group in the training set and the generated molecules.

S5 Accuracy of P Value Predictions

Following Koczor-Benda *et al.* [4], 65% of the molecules from the THz database were used for training (1,752), 20% were used for testing (546) and the remaining molecules were used for validation (438). For the Thiol database and Iteration 6, converged DFT calculations were obtained for 131 and 197 molecules, respectively,

out of the 200 randomly selected molecules. These are used as test sets for the KRR and PaiNN models trained on the THz training set.

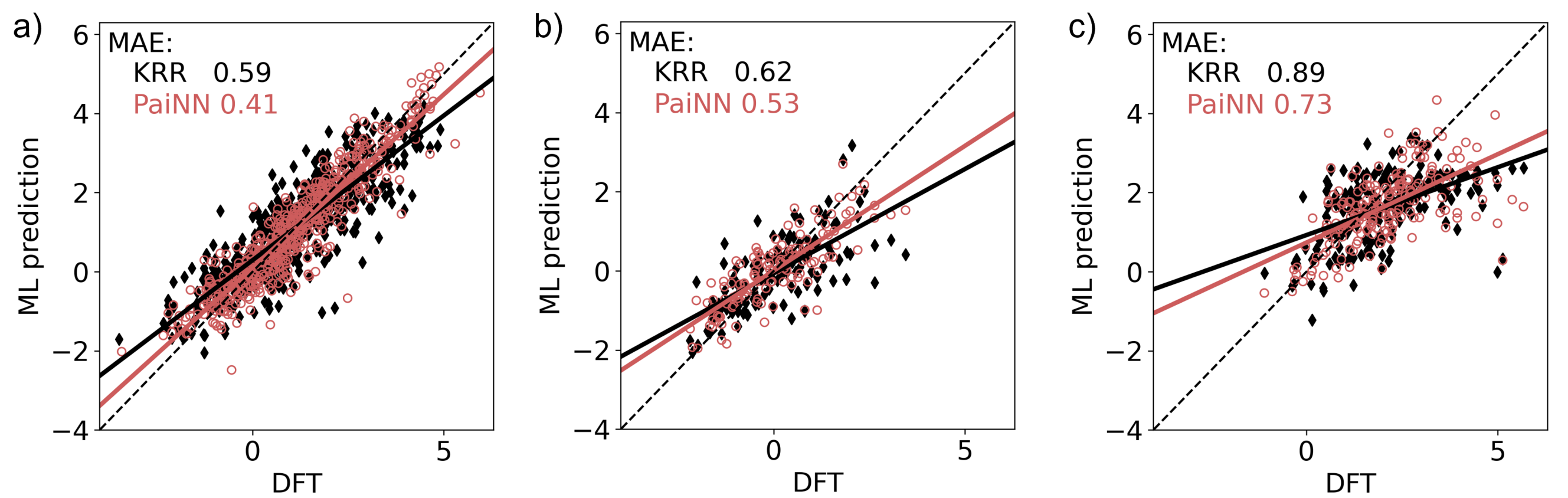

Figure S6. Accuracy of KRR (black diamonds) and PaiNN (pink circles) predictions for the P value for test molecules from the a) THz database b) Thiol database c) Molecules generated in Iteration 6. In the case of the PaiNN model, predictions are based on DFT-optimized molecular structures.

S6 Retraining the PaiNN Property Predictor

Next, a committee of 5 PaiNN models was trained using different train/validation sets (80%/10% split) and random seeds, and the same test set (10%) across the 5 models (Figure S7). MAE values on the test sets in Figure S7 are slightly different from Figure S6 due to the different splitting of data. We find that the uncertainty of the predictions does not correlate with the accuracy of the predictions in any of these cases. The uncertainty of the committee tends to be very small compared to the range of P values and the prediction errors for most molecules.

Finally, randomly selected molecules from the Thiol database and Iteration 6 were added to the training set, to improve the accuracy of predictions for generated molecules (Figure S8).

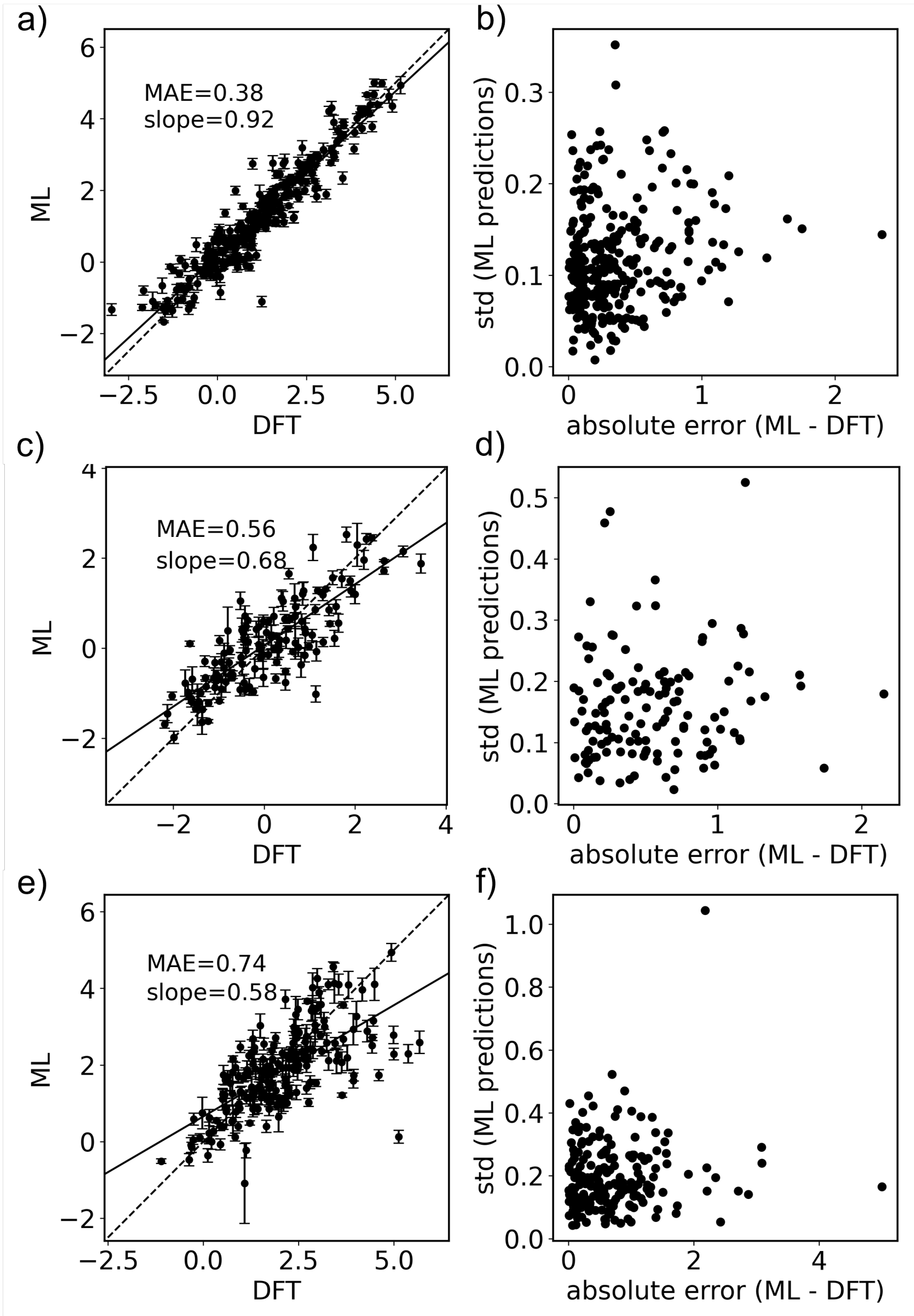

Figure S7. P value predictions of a committee of 5 PaiNN models trained on the THz set for test molecules from a) the THz database, c) the Thiol database, and e) Biasing Iteration 6. For all test molecules, the mean average and standard deviation of P values predicted by the 5 PaiNN models is shown. Standard deviation of the predictions versus absolute error of the mean of the predictions for test molecules from b) the THz database, d) the Thiol database, and f) Biasing Iteration 6.

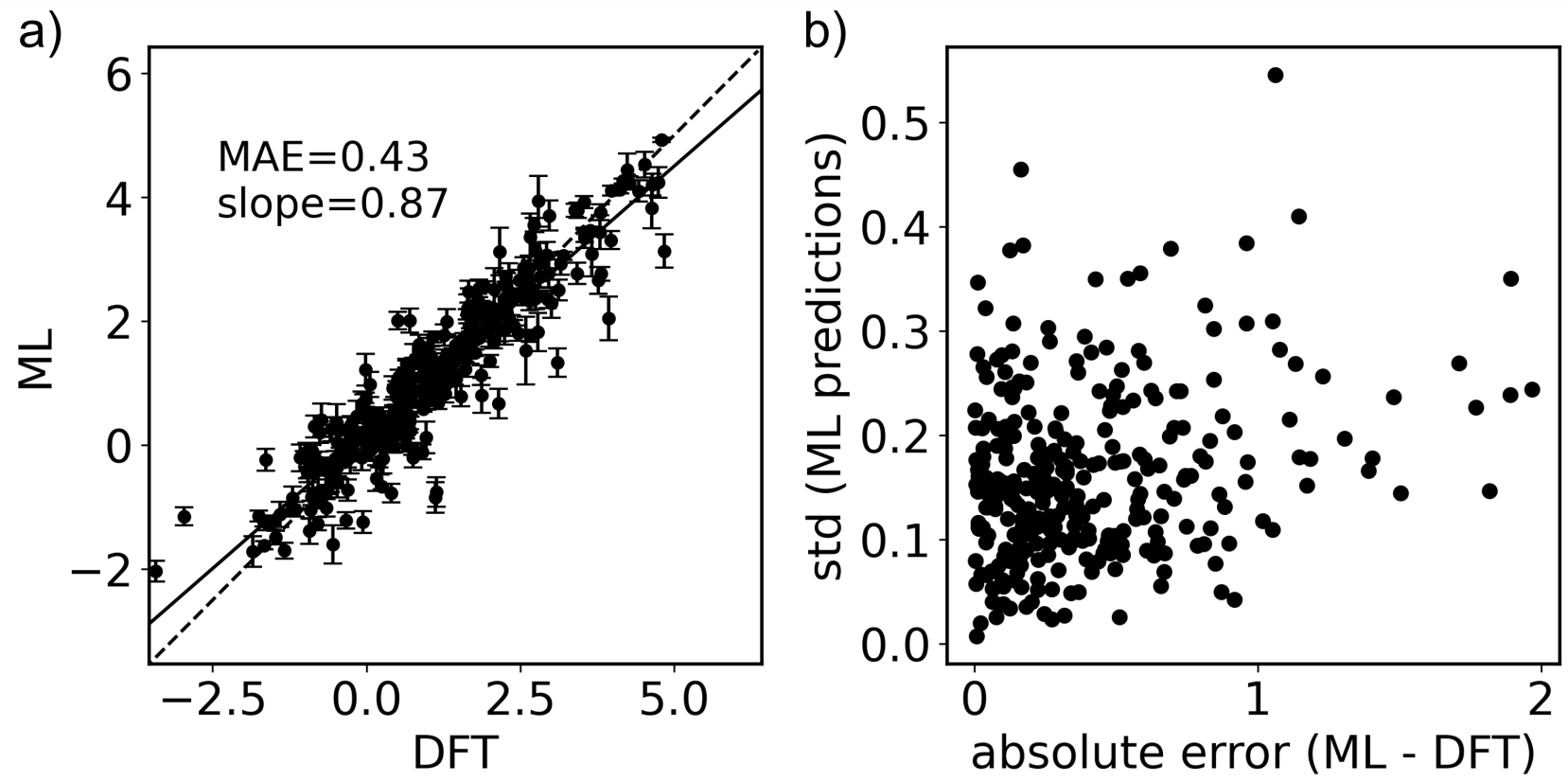

Figure S8. a) P value predictions on test molecules by a committee of 5 PaiNN models trained on the combined THz set plus randomly selected molecules from the Thiol database and Biasing Iteration 6. For all test molecules, the mean average and standard deviation of P values predicted by the 5 PaiNN models is shown. b) Standard deviation of the predictions versus absolute error of the mean of the predictions for test molecules.

S7 Analysis of PaiNN Predictions for Generated Molecules

P values were predicted for all training and generated molecules using the retrained committee of PaiNN models, and the distribution of P values were plotted according to the absence or presence of specific functional groups identified by Koczor-Benda *et al.*[4] as correlating with high P values (Fig. S9). The presence of functional groups b), c), d) and e) show a correlation with higher P values, while the presence of a) and f) does not affect the P values apart from the training set. This suggests that the latter functional groups are themselves not correlated with higher P values, but they rather occur together with other molecular features that promote higher P values in the commercial thiol database (used for training).

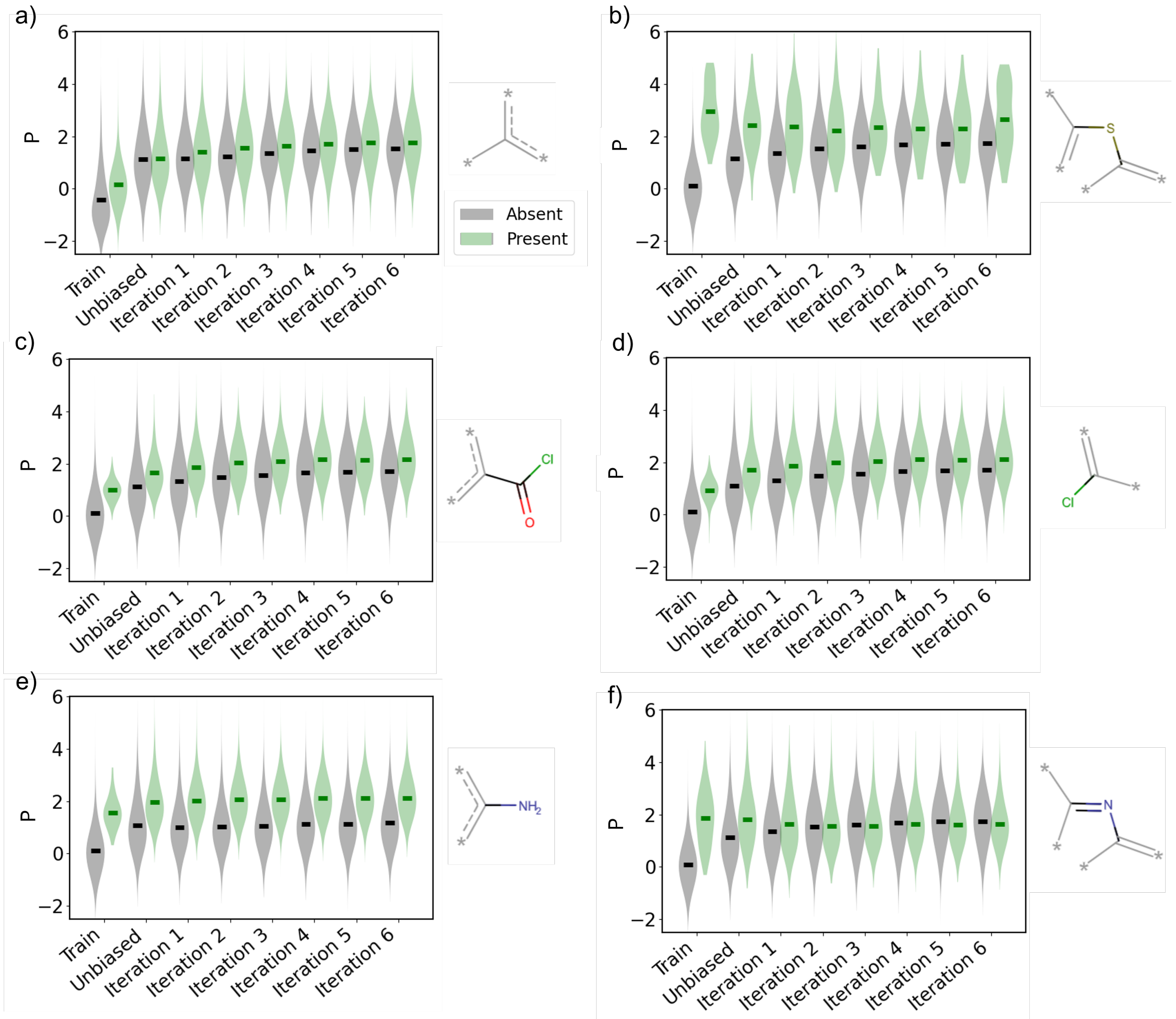

Figure S9. Distribution and mean of P values predicted by PaiNN for molecules in which the functional group depicted is absent (gray) or present (green).

S8 Chemical Space Mapping of Generated Molecules

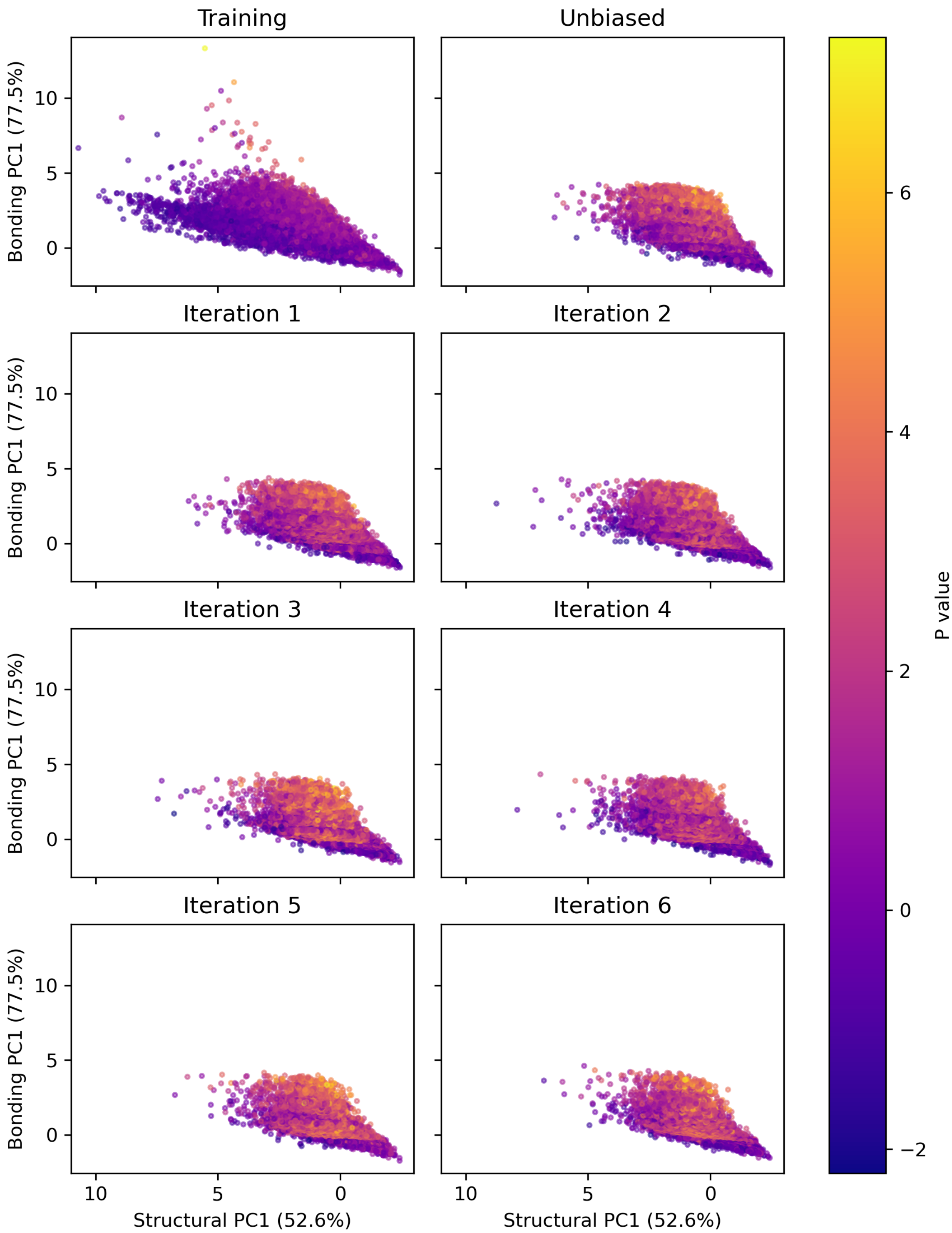

Figure S10. Latent chemical space plots of training molecules, and molecules generated in subsequent biasing iterations, colored by their PaiNN-predicted P values.

S9 Retrosynthetic Paths and Vibrational Spectra of Candidate Molecules

Table SV details how many molecules satisfied the $P_{\mathrm{PaiNN}} \geq 4.25$ criterion for each database, as predicted from a committee of PaiNN models. Table SVI details the properties of the top generated molecules with solved retrosynthetic pathways using the AiZynthFinder[6] software and Figures S11–S17 show their top retrosynthesis paths based on a stock from compounds available within the ZINC[7] database and a policy[8] trained on US patent office data[9], as available within AiZynthFinder.

Figures S18–S21 detail the vibrational spectra and properties of select candidate molecules, with their IDs taken from Table SVI.

Database	Number of Molecules
Unbiased	128
Bias 1	192
Bias 2	175
Bias 3	140
Bias 4	123
Bias 5	112
Bias 6	141

Table SV. Number of molecules within each database of generated molecules satisfying $P_{PaiNN} \geq 4.25$.

Molecule ID	Iteration	P_{DFT}	P_{PaiNN}	P_{KRR}	SCScore	Cluster	SMILES
27354	4	7.88	5.56	3.23	3.11	C2	Nc1ccc(cc1)Nc1cnc(cc1S)N
44707	3	5.89	4.66	2.09	3.54	C2	OCc1nc(N)ccc1Nc1ncc(c(c1)CS)N
14048	5	5.37	4.44	2.31	1.91	C5	Nc1nc(N)c(c(c1N)S)N
12789	6	5.29	4.39	2.31	1.91	C5	Nc1nc(N)c(c(c1N)S)N
13796	4	5.29	4.47	2.31	1.91	C5	Nc1nc(N)c(c(c1N)S)N
8518	2	5.29	4.36	2.31	1.91	C5	Nc1nc(N)c(c(c1N)S)N
43020	6	5.23	4.83	3.33	2.87	C2	Oc1cc(ccc1N)Oc1ccc(c(c1)S)N
38431	4	5.2	4.5	2.07	1.64	C5	Nc1c(N)c(N)c(c(c1N)S)N
34759	6	5.14	4.39	2.99	2.16	C5	Nc1cc(S)c(cc1N)N
9160	5	5.14	4.48	2.99	2.16	C5	Nc1cc(S)c(cc1N)N
18986	4	5.12	4.39	3.29	2.14	C5	SC(=S)Nc1ccc(c(c1)N)N
22423	6	4.99	4.35	2.07	1.64	C5	Nc1c(N)c(N)c(c(c1N)S)N
26622	1	4.83	4.57	2.07	1.64	C5	Nc1c(N)c(N)c(c(c1N)S)N
4030	2	4.8	4.35	2.84	2.29	C5	Nc1c(N)cc(c(c1Br)S)N
43713	5	4.78	4.73	3.09	2.92	C2	COc1cc(O)c(cc1N)C(=O)c1cc(N)ccc1S
11249	3	4.77	4.46	2.31	1.91	C5	Nc1nc(N)c(c(c1N)S)N
4411	2	4.77	4.67	2.99	2.16	C5	Nc1cc(S)c(cc1N)N
23355	1	4.77	4.39	2.31	1.91	C5	Nc1nc(N)c(c(c1N)S)N
43114	5	4.73	4.27	2.87	2.94	C2	Nc1ccc2c(c1)ccc(c2)Nc1ccc(cc1S)C(=O)O
43881	4	4.12	4.54	2.81	2.85	C2	Nc1ccc(c(c1)Nc1cccc(c1C)N)S
18261	6	3.87	4.43	3.27	2.28	C5	Nc1ccc(cc1NC(=S)S)N
23538	3	3.80	4.60	2.65	2.53	C5	Oc1cc(S)c(cc1N)S(=O)(=O)N
31446	5	3.61	5.01	3.63	3.22	C2	O=Cc1c(ccc(c1N)N)c1ccc(c(c1)S)N
27425	1	3.53	4.63	2.57	2.98	C2	Nc1ccc(cc1)n1c(S)nc2c1cc(N)cc2
17848	6	3.46	4.35	2.21	1.90	C5	Nc1c(Br)c(N)c(c(c1N)S)N
18617	4	3.46	4.78	2.21	1.90	C5	Nc1c(Br)c(N)c(c(c1N)S)N
13331	2	3.45	4.57	2.62	2.11	C5	Nc1cc(N)c(c(c1N)S)N
25012	3	3.24	4.52	2.21	1.90	C5	Nc1c(Br)c(N)c(c(c1N)S)N
12734	2	3.24	4.30	2.21	1.90	C5	Nc1c(Br)c(N)c(c(c1N)S)N
19179	5	3.23	4.35	2.21	1.90	C5	Nc1c(Br)c(N)c(c(c1N)S)N
31762	2	3.04	4.63	3.15	3.10	C2	Nc1ccc(cc1)C(=O)Nc1cnc(cc1S)N
33480	3	2.85	4.36	2.07	1.64	C5	Nc1c(N)c(N)c(c(c1N)S)N
45647	6	1.65	4.33	1.84	3.35	C2	NC(=O)COC(=O)c1cc(N)c(cc1Nc1ccccc1S)O
39452	3	1.40	4.67	2.43	3.03	C2	Nc1cc(C)c(c(c1)S)Nc1nnc(s1)N

Table SVI. Properties of the top generated molecules with solved retrosynthetic pathways.

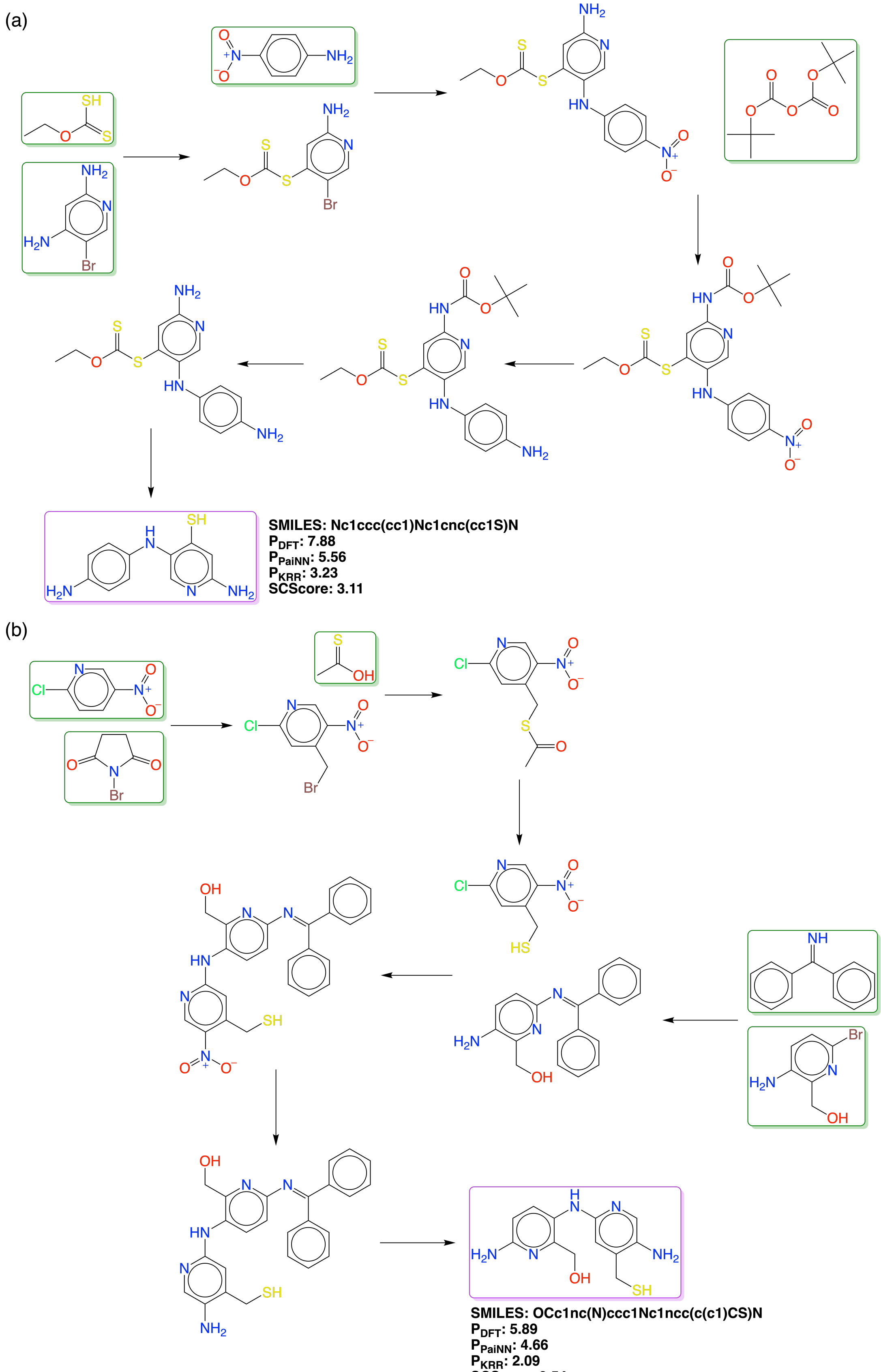

Figure S11. Top retrosynthetic paths for molecules with SMILES strings (a) Nc1ccc(cc1)Nc1cnc(cc1S)N and (b) OCc1nc(N)ccc1Nc1ncc(c(c1)CS)N. The final molecules are shown in purple boxes and precursors available within the ZINC database are shown in green boxes.

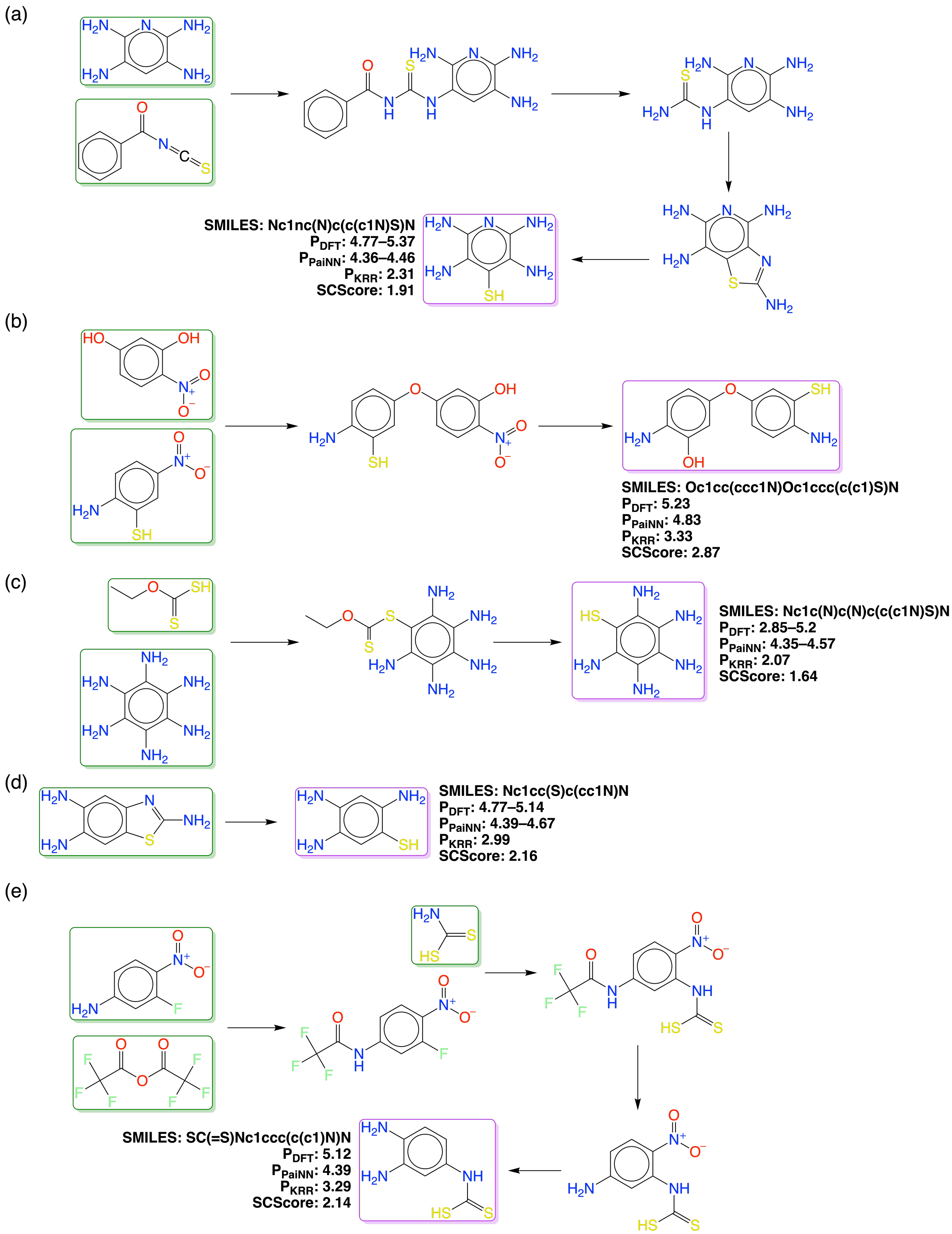

Figure S12. Top retrosynthetic paths for molecules with SMILES strings (a) Nc1nc(N)c(c(c1N)S)N, (b) Oc1cc(ccc1N)Oc1ccc(c(c1)S)N, (c) Nc1c(N)c(N)c(c(c1N)S)N, (d) Nc1cc(S)c(cc1N)N, and (e) SC(=S)Nc1ccc(c(c1)N)N. The final molecules are shown in purple boxes and precursors available within the ZINC database are shown in green boxes.

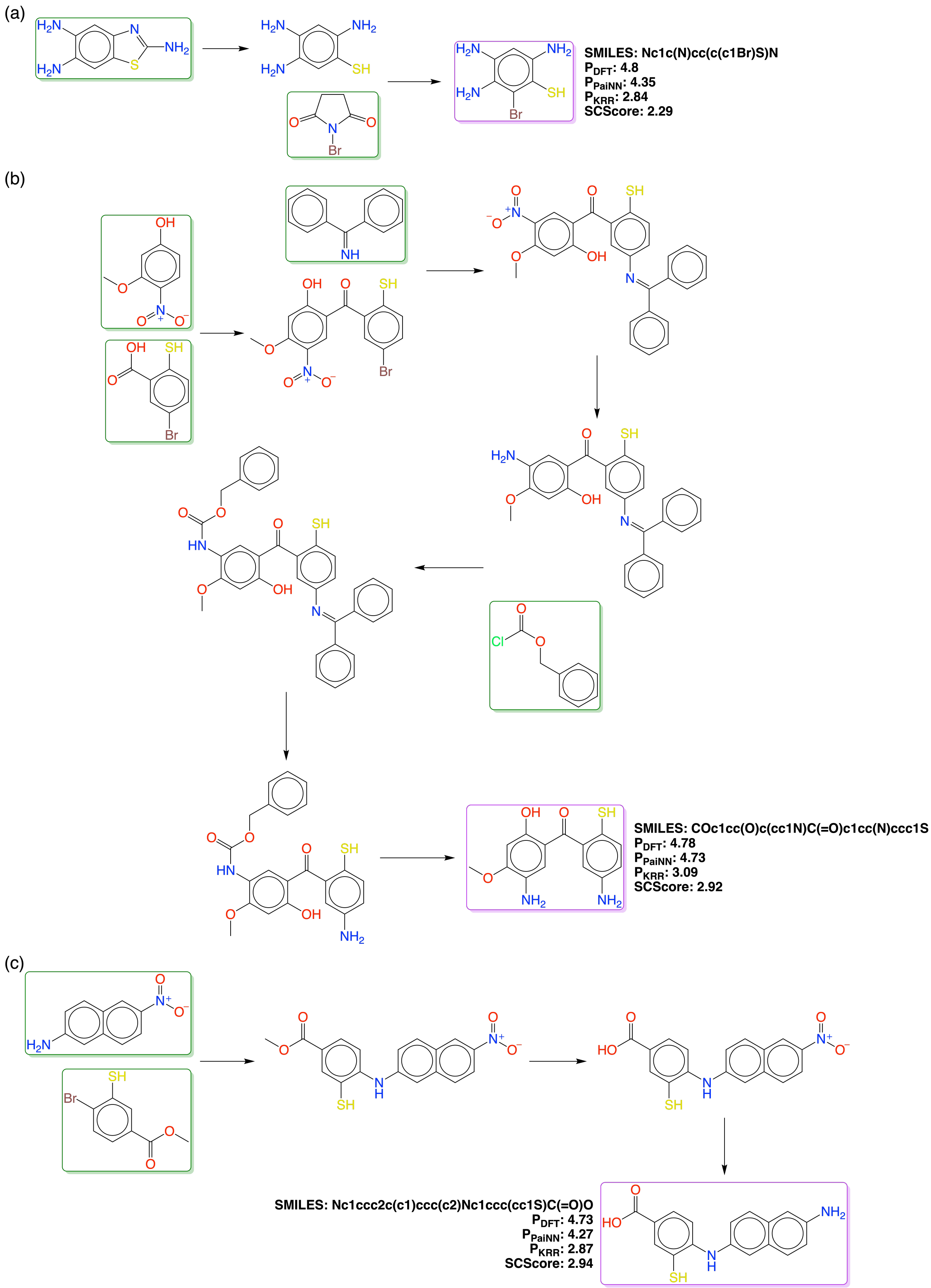

Figure S13. Top retrosynthetic paths for molecules with SMILES strings (a) Nc1c(N)cc(c(c1Br)S)N, (b) COc1cc(O)c(cc1N)C(=O)c1cc(N)ccc1S, and (c) Nc1ccc2c(c1)ccc(c2)Nc1ccc(cc1S)C(=O)O. The final molecules are shown in purple boxes and precursors available within the ZINC database are shown in green boxes.

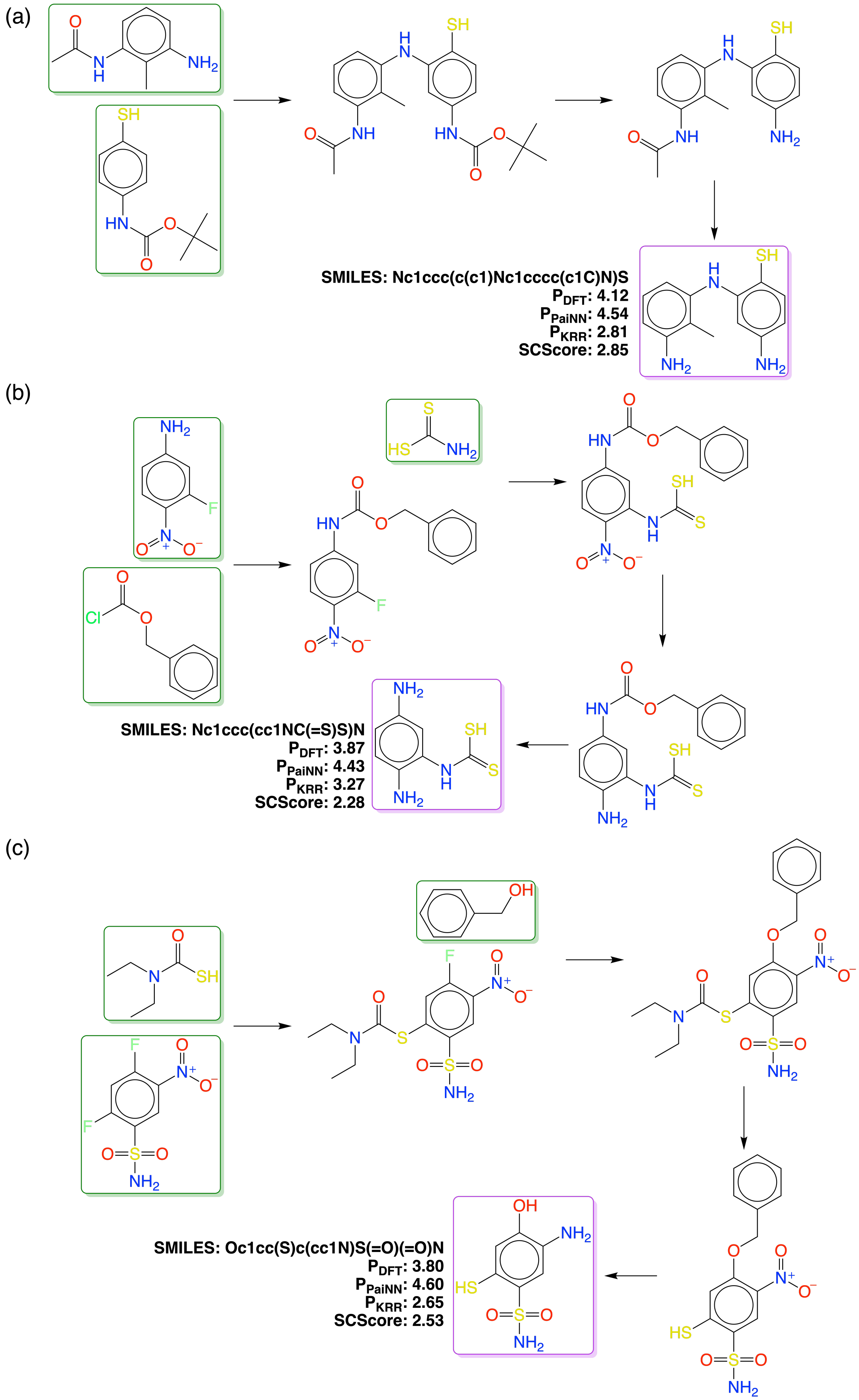

Figure S14. Top retrosynthetic paths for molecules with SMILES strings (a) Nc1ccc(c(c1)Nc1cccc(c1C)N)S, (b) Nc1ccc(cc1NC(=S)S)N, and (c) Oc1cc(S)c(cc1N)S(=O)(=O)N. The final molecules are shown in purple boxes and precursors available within the ZINC database are shown in green boxes.

(a)

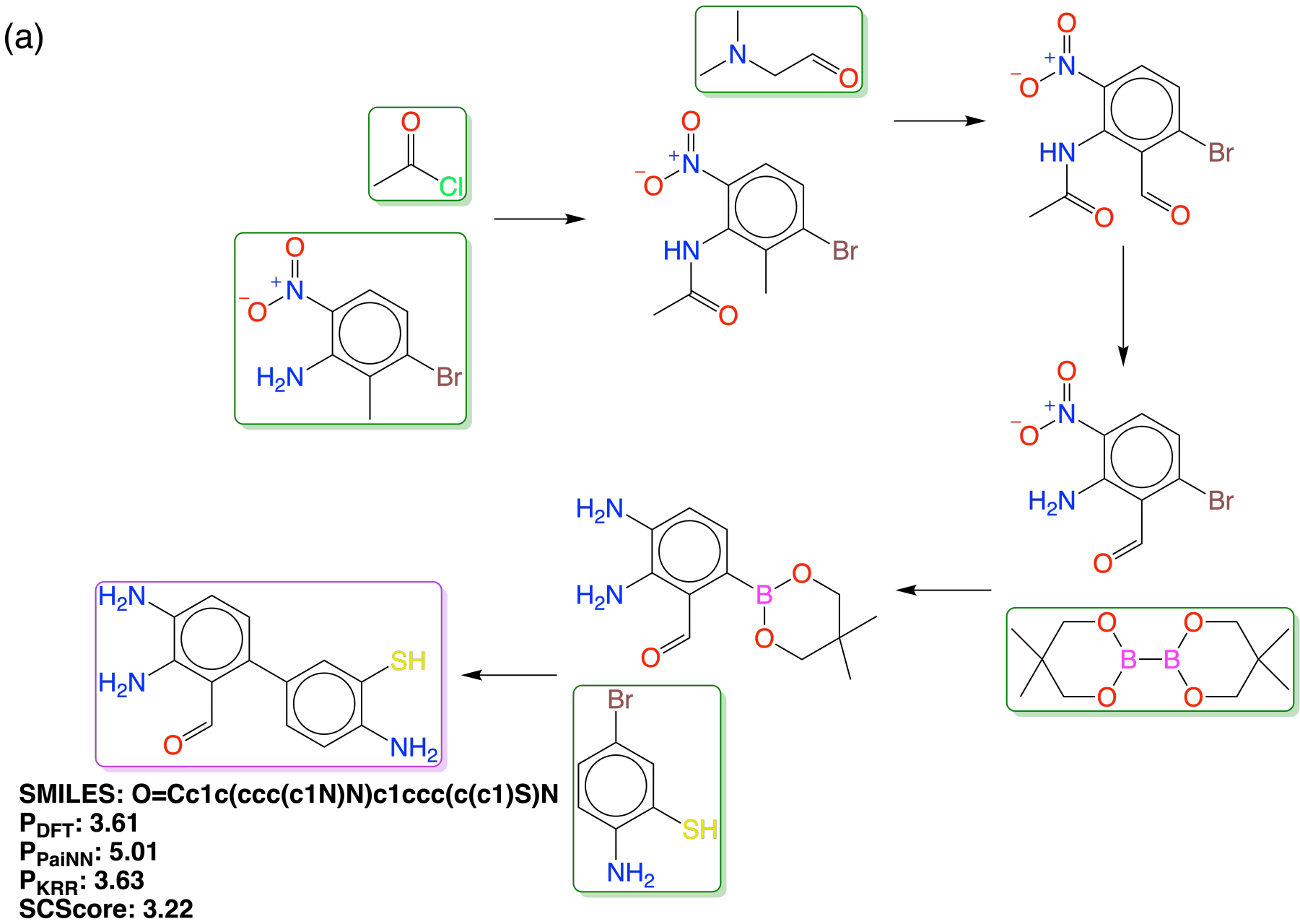

(b)

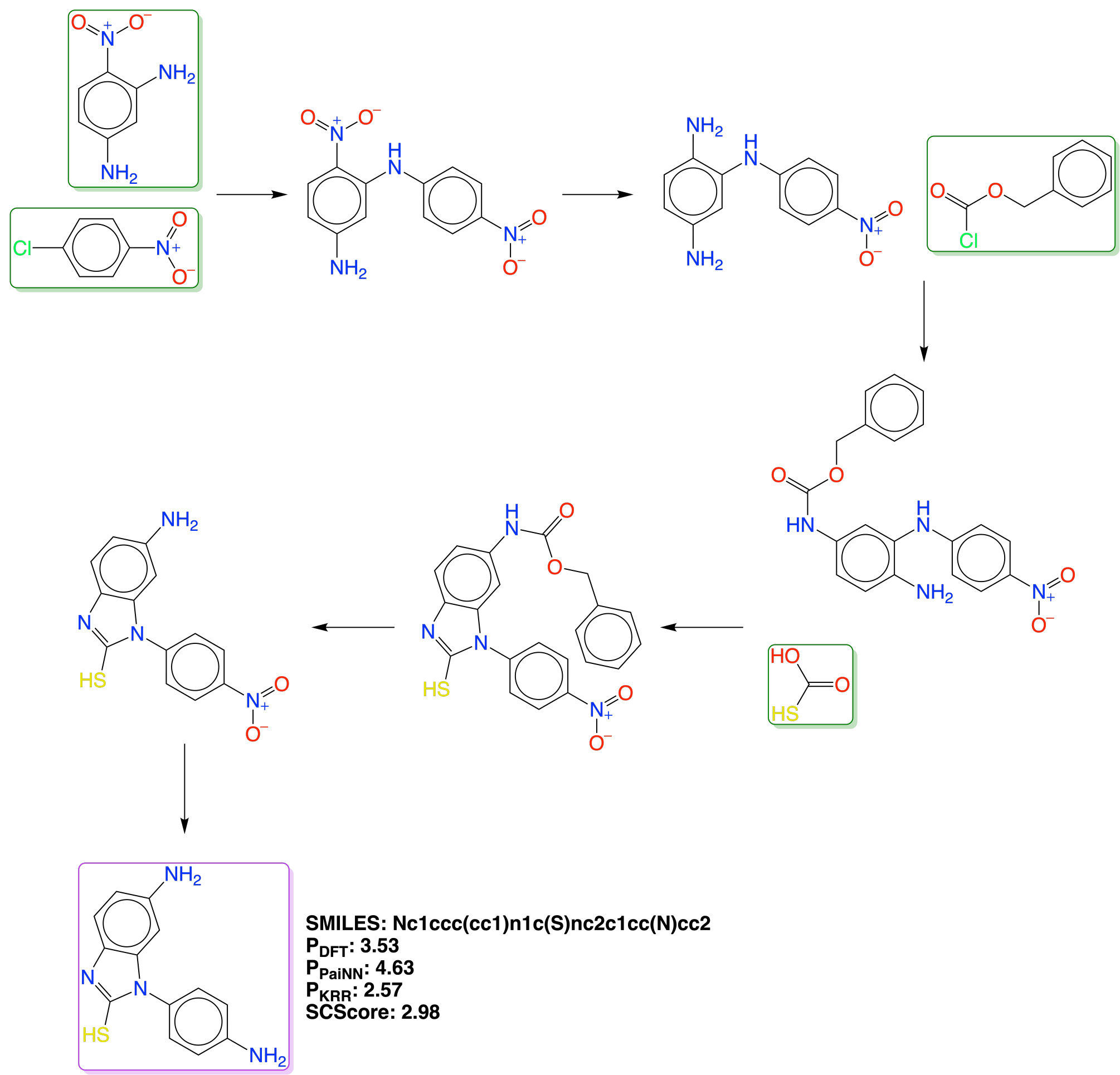

Figure S15. Top retrosynthetic paths for molecules with SMILES strings (a) O=Cc1c(ccc(c1N)N)c1ccc(c(c1)S)N and (b) Nc1ccc(cc1)n1c(S)nc2c1cc(N)cc2. The final molecules are shown in purple boxes and precursors available within the ZINC database are shown in green boxes.

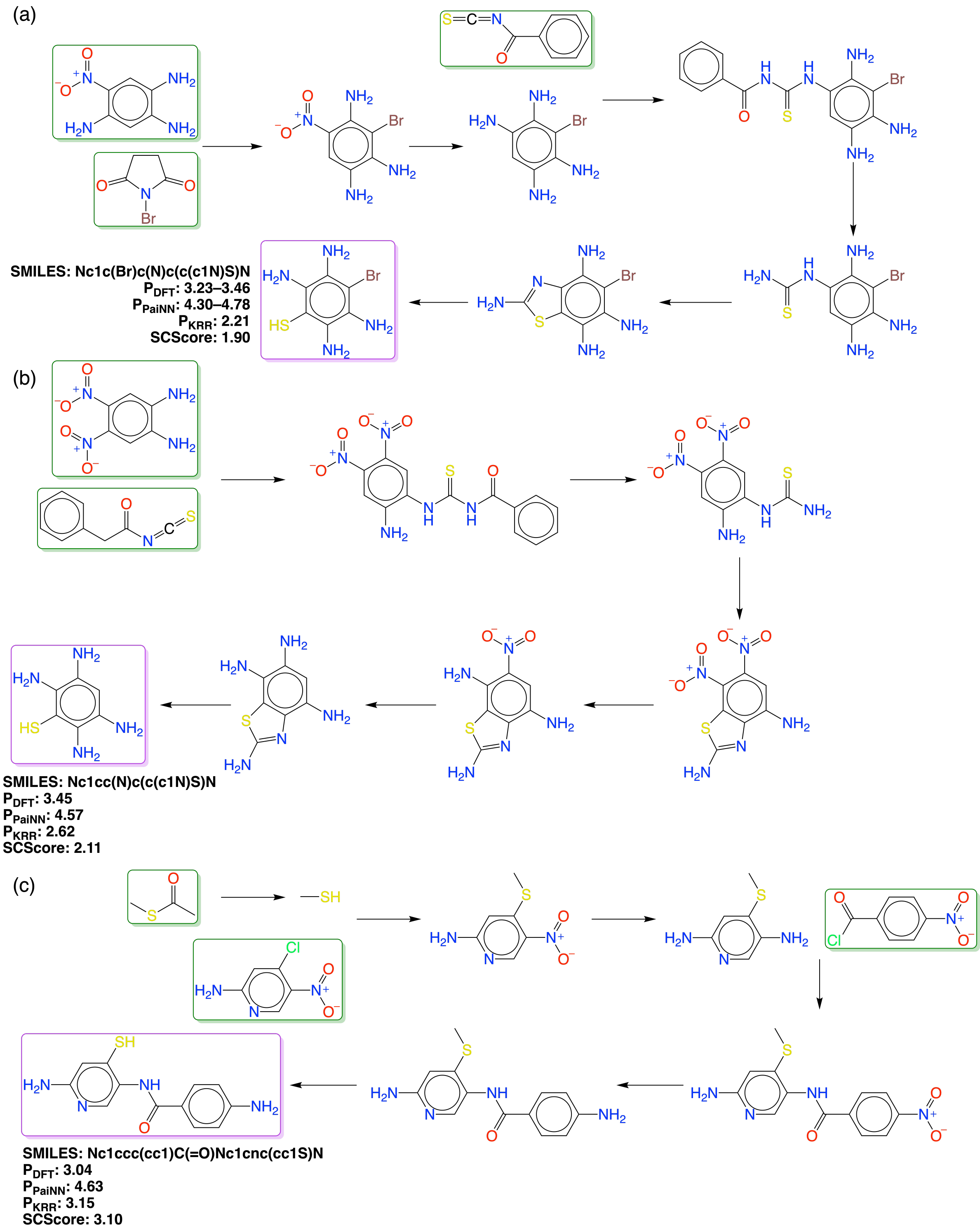

Figure S16. Top retrosynthetic paths for molecules with SMILES strings (a) Nc1c(Br)c(N)c(c(c1N)S)N, (b) Nc1cc(N)c(c(c1N)S)N, and (c) Nc1ccc(cc1)C(=O)Nc1cnc(cc1S)N. The final molecules are shown in purple boxes and precursors available within the ZINC database are shown in green boxes.

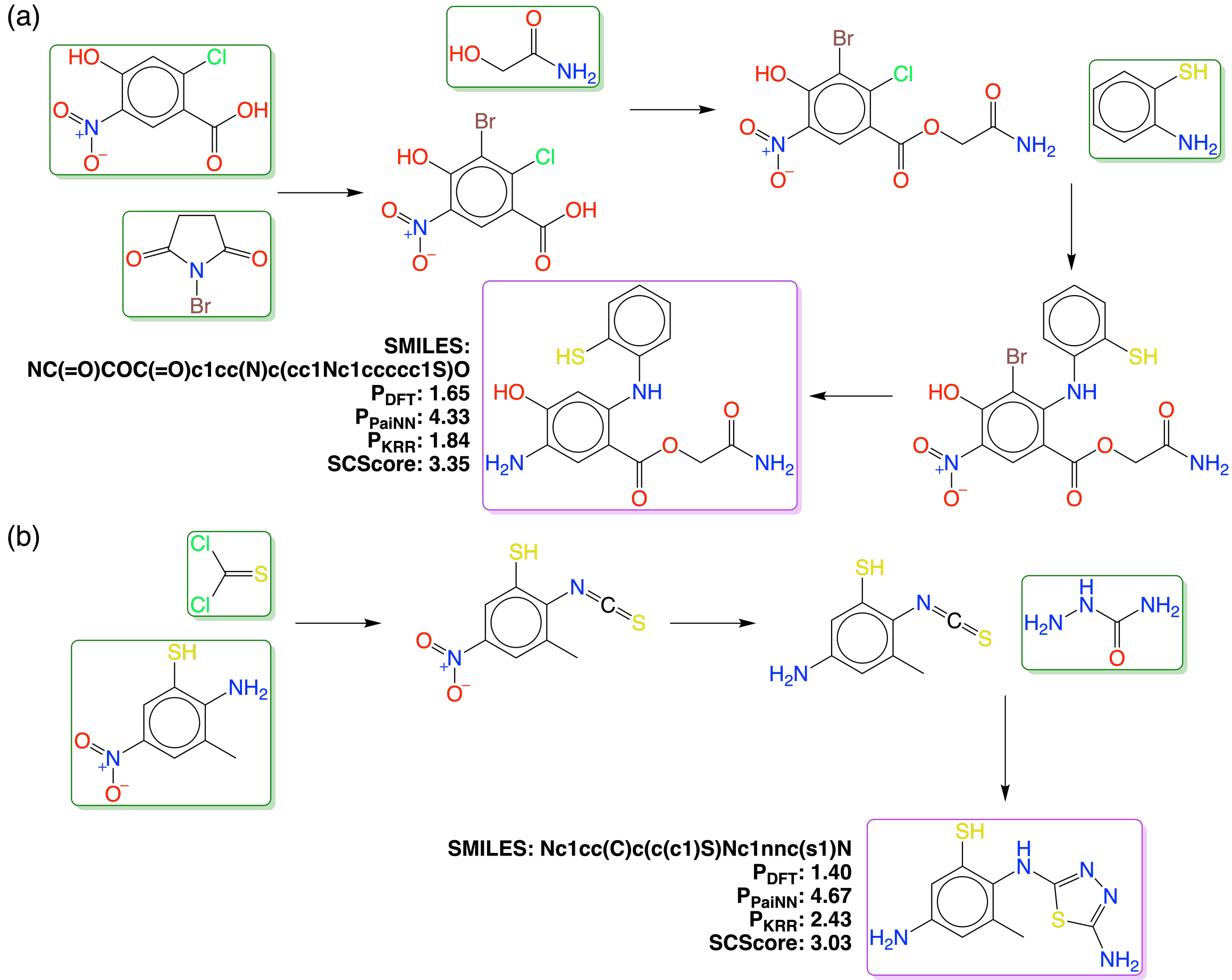

Figure S17. Top retrosynthetic paths for molecules with SMILES strings (a) NC(=O)COC(=O)c1cc(N)c(cc1Nc1ccccc1S)O and (b) Nc1cc(C)c(c(c1)S)Nc1nnc(s1)N. The final molecules are shown in purple boxes and precursors available within the ZINC database are shown in green boxes.

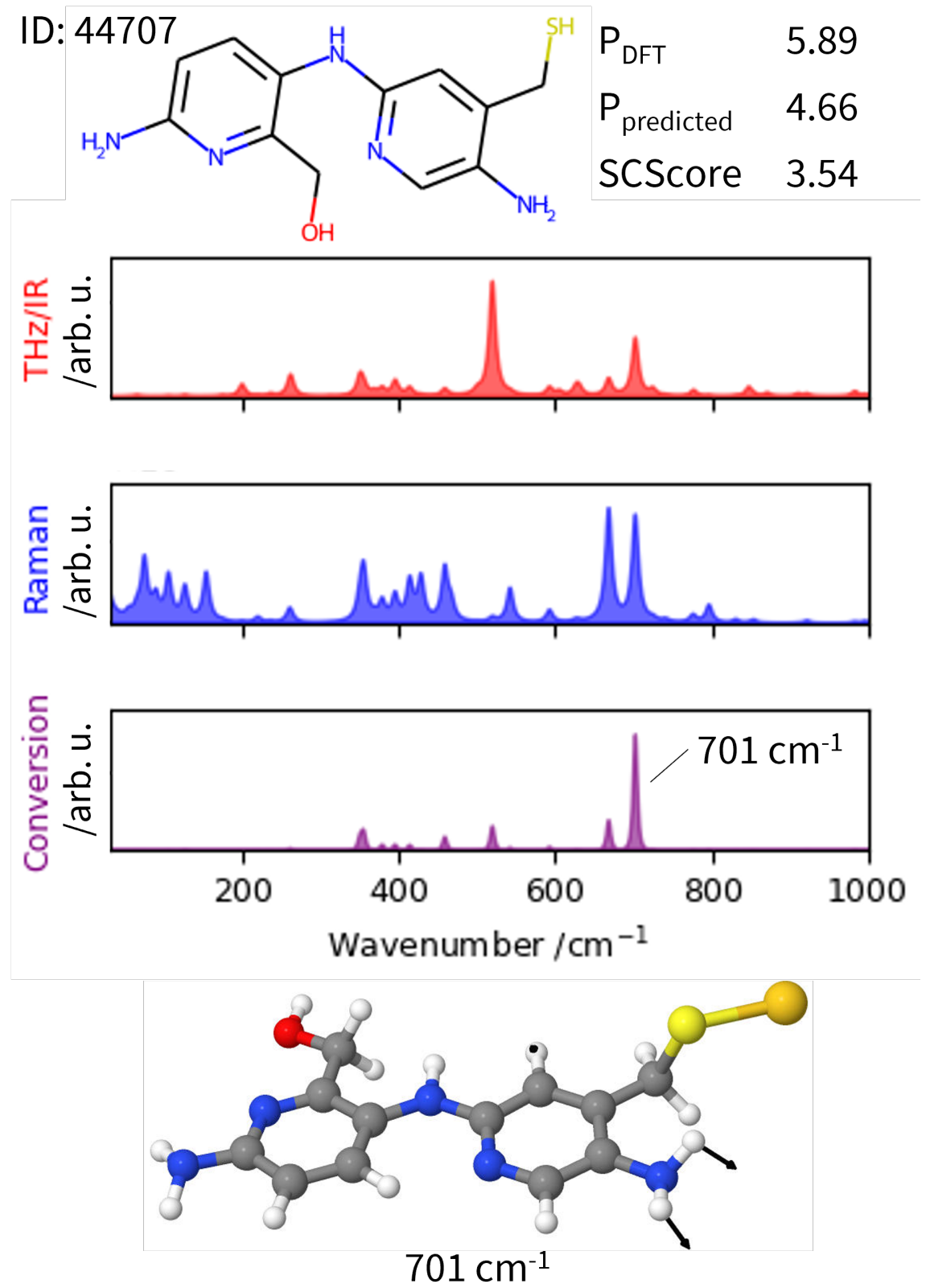

Figure S18. Vibrational spectra and properties of candidate molecule with ID 44707.

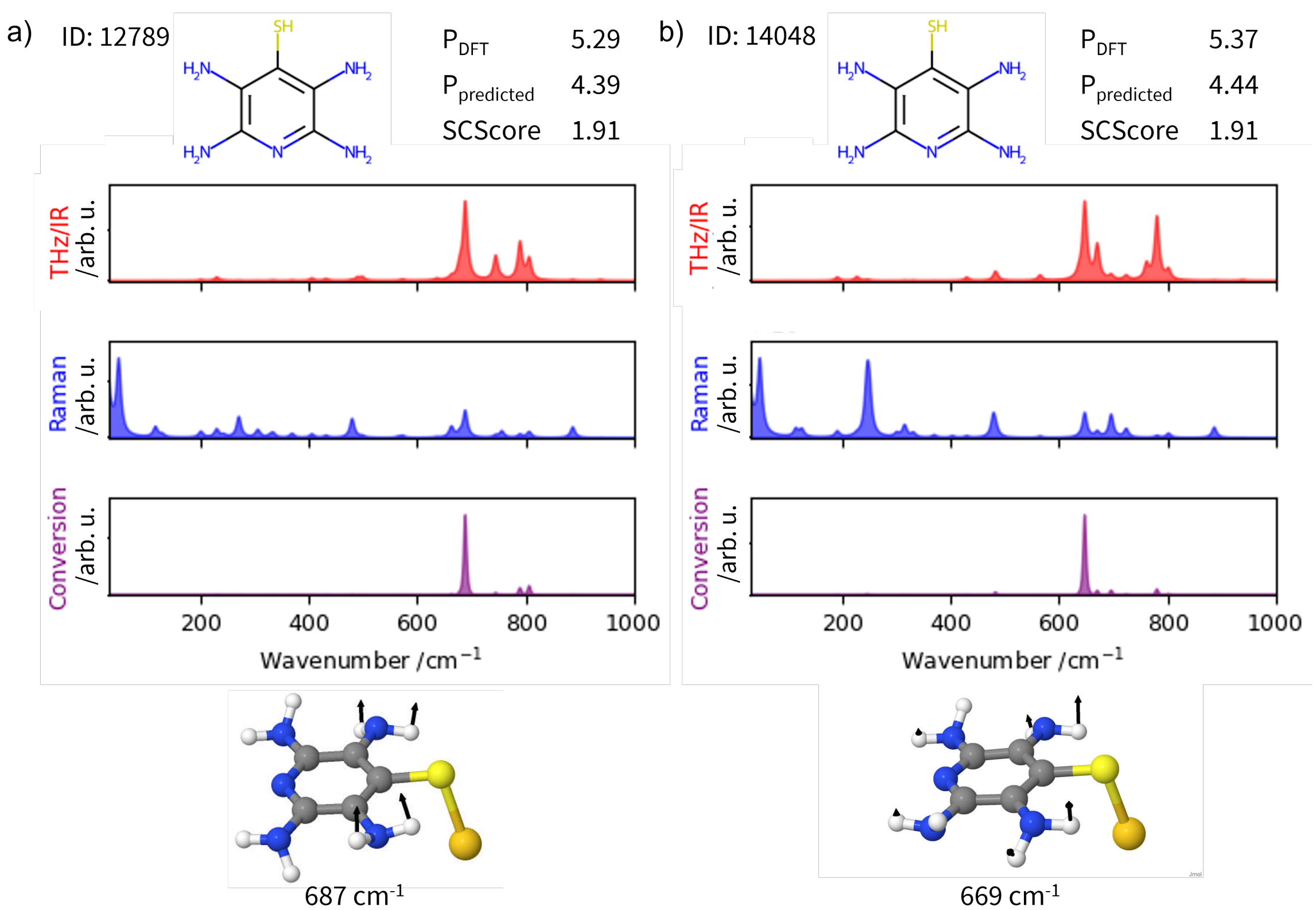

Figure S19. Vibrational spectra and properties of candidate molecules with IDs a) 12789 and b) 14048.

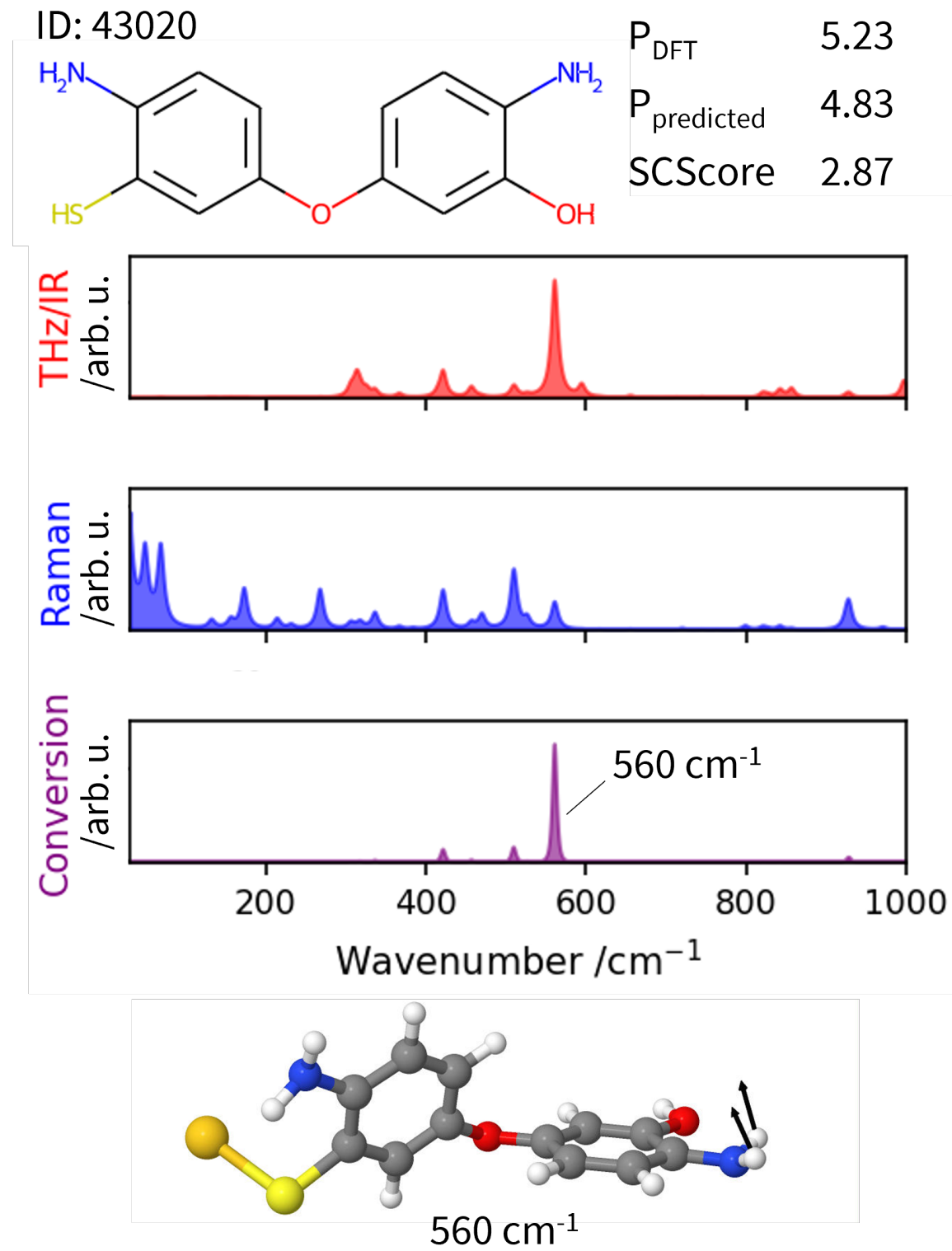

Figure S20. Vibrational spectra and properties of candidate molecule with ID 43020.

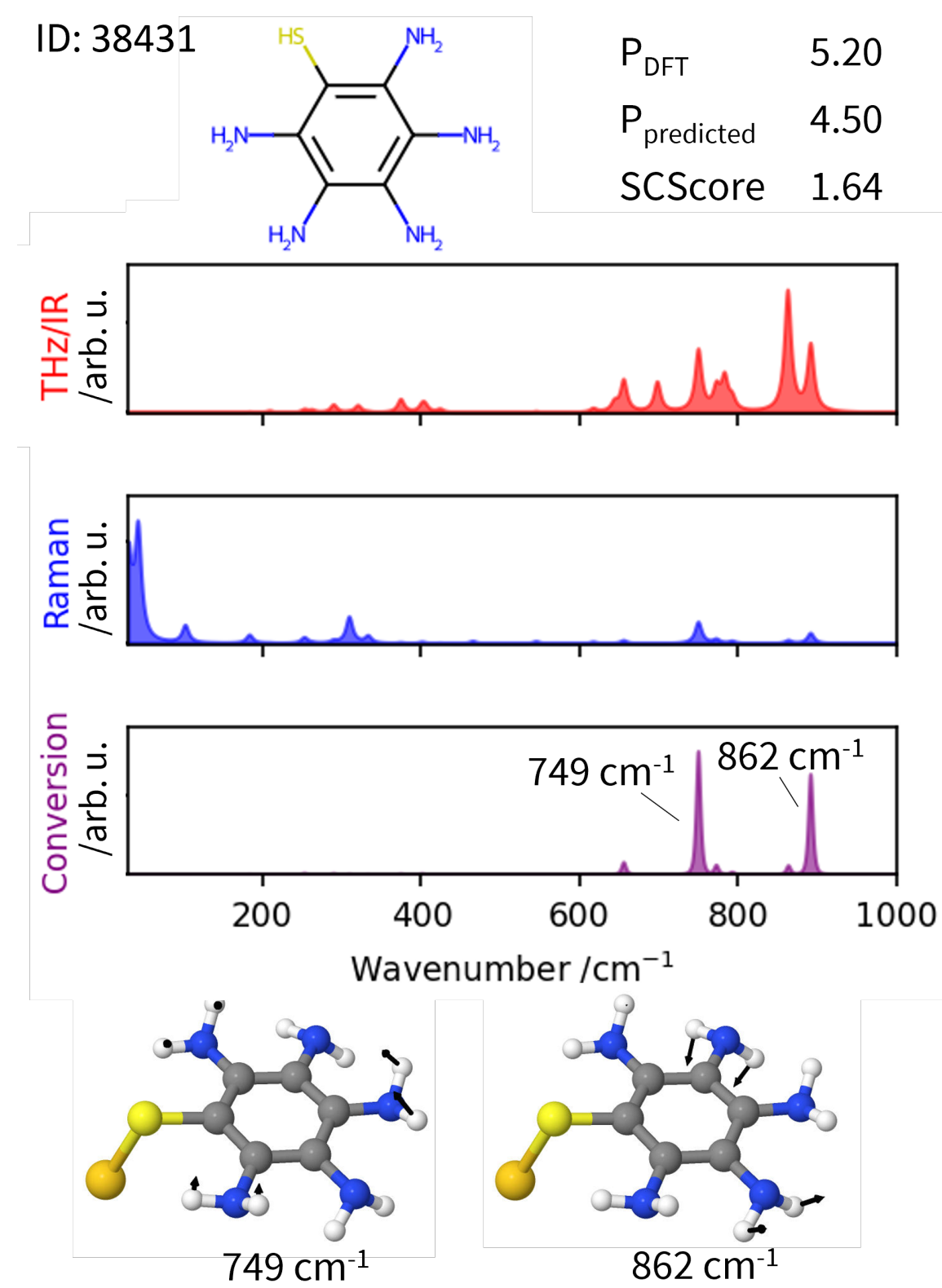

Figure S21. Vibrational spectra and properties of candidate molecule with ID 38431.

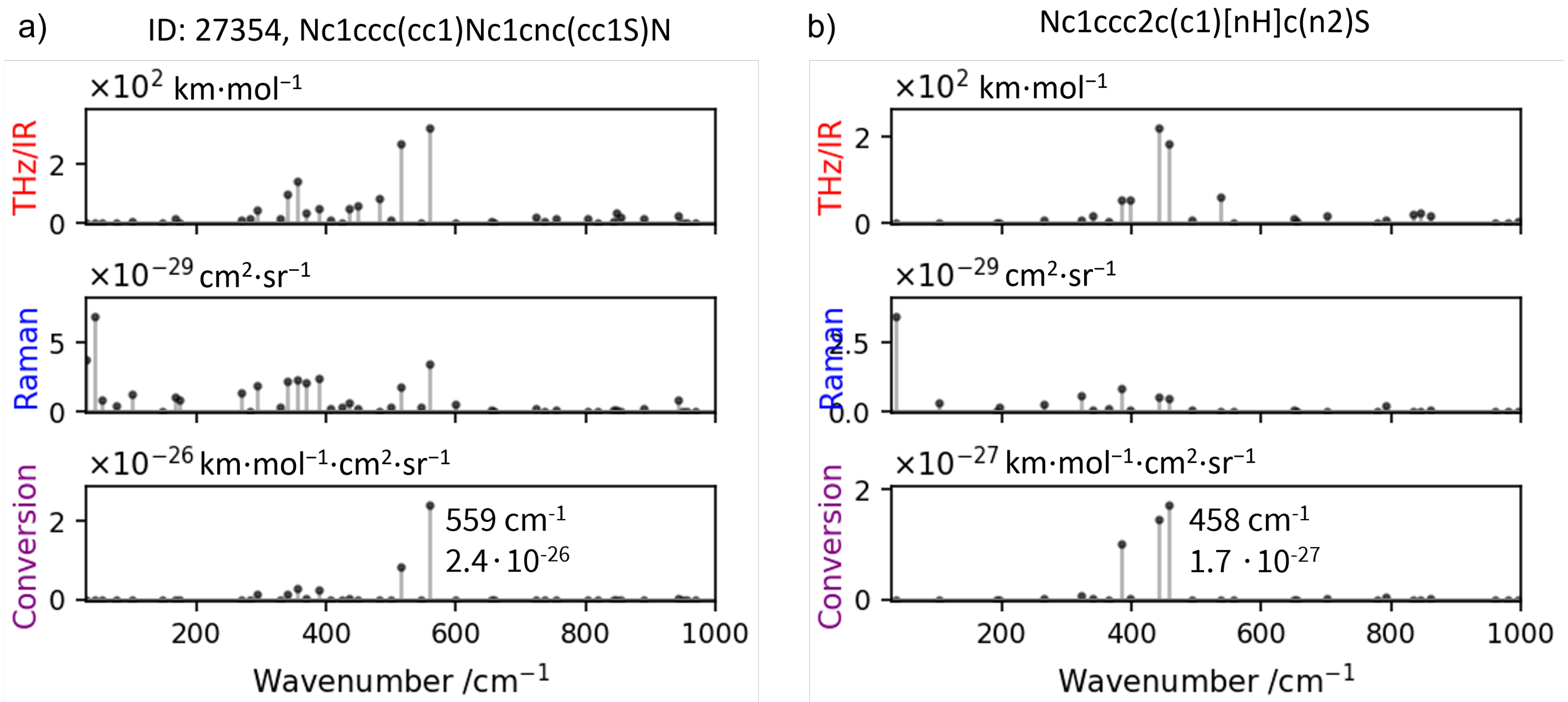

Figure S22. Comparison of vibrational properties between a) the top candidate molecule with ID 27354 and b) 5-Amino-2-mercaptobenzimidazole, available from Molecular Vibration Explorer[5].

S10 Structural Validation of Generated Molecules

Most 3D generative models of molecules have so far been trained on DFT-optimized structures[3,10,11]. To assess the effect of using the semi-empirical xTB method instead of DFT on the accuracy of the generated structures, subsequent xTB and DFT optimizations were performed on randomly selected molecules from the unbiased generation. As shown in Fig. S22, the distribution of root-mean-square deviation (RMSD) values between generated and xTB-optimized structures is narrower and is centered at a smaller value than previously reported RMSD values between DFT-optimized structures and structures generated using G-SchNet (and trained on the OE62[12] dataset).[3] This is likely due to the larger maximum size of generated molecules (100 atoms) set by Westermayr *et al.*[3] compared to this work (60 atoms). The RMSD values of generated structures compared to DFT-optimized structures are somewhat larger than compared to xTB-optimized structures, as shown in Fig. S23a, which is to be expected. Since the PaiNN predictor relies on 3D molecular structures, we are able to test how the structural relaxation of the generated molecules affects the property predictions. We compared predictions of the same PaiNN model on unrelaxed generated structures and the corresponding DFT-optimized structures for the randomly selected molecules from Iteration 6. As shown in Fig. S23b, using the unrelaxed generated structures for P value prediction yields significant changes in the predicted values and would yield even further underestimation of high P values in the biasing workflow.

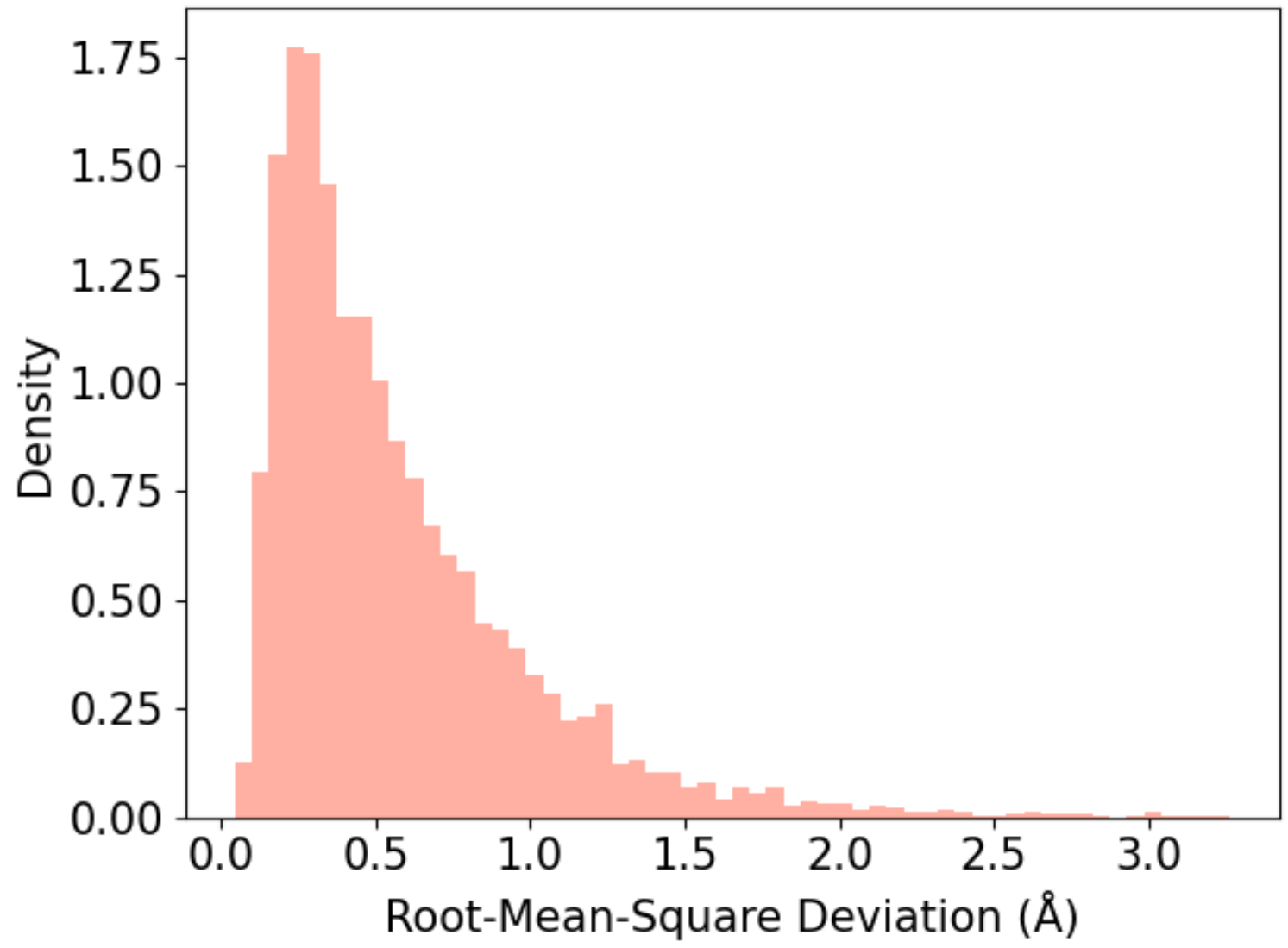

Figure S23. Distribution of the root-mean-square deviations of atomic positions between the molecules generated using an unbiased G-SchNet model and the same molecules subsequently optimized using the reference xTB method.

For unrelaxed generated structures, when exchanging the thiol-hydrogen to a gold atom, a fixed sulfur-gold bond length of 2.88 Å was applied, keeping all other internal coordinates unchanged from the generated structure.

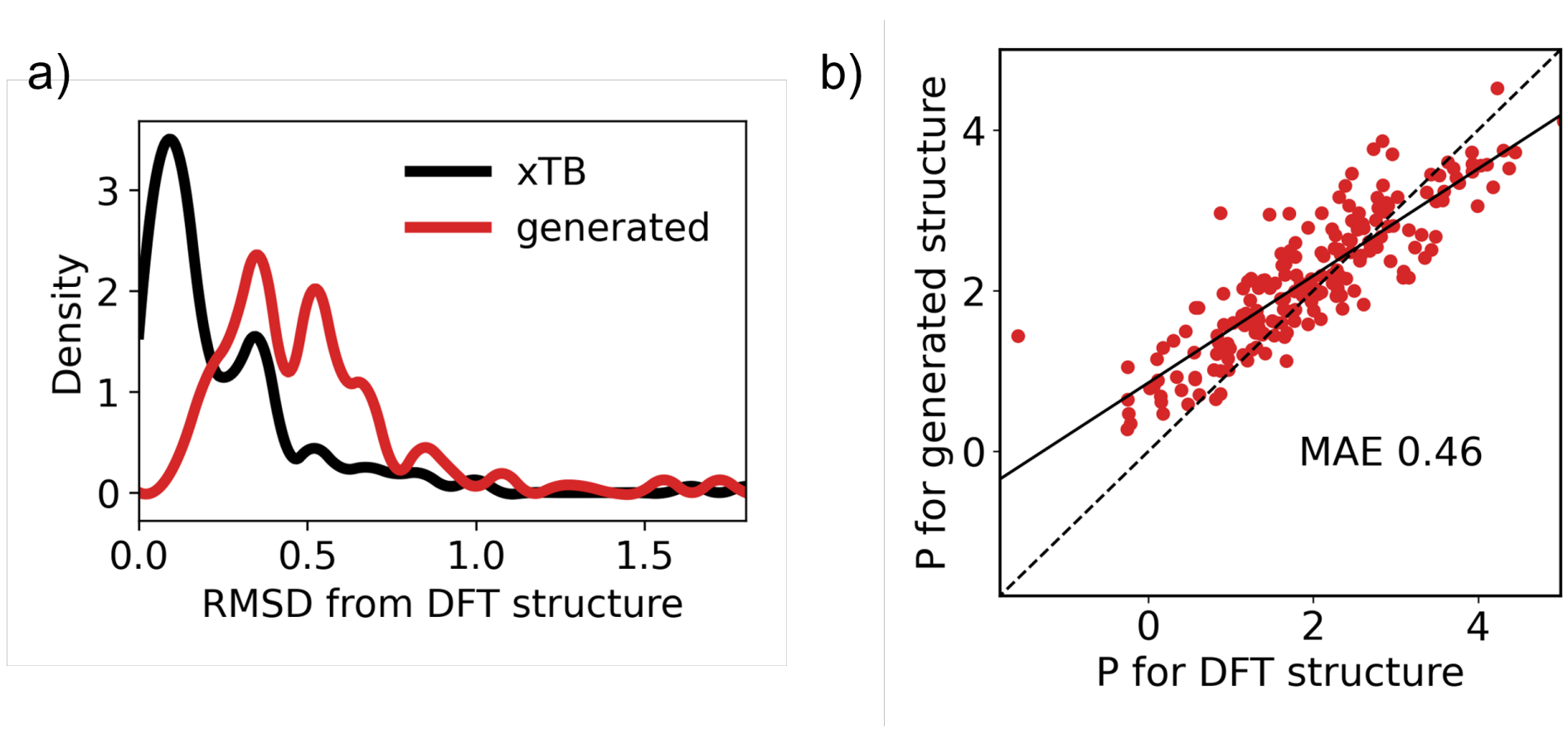

Figure S24. a) Distributions of the root-mean-square deviations (RMSDs) of atomic positions between DFT structures and the 3D structures generated using an unbiased G-SchNet model and the same molecules subsequently optimized using xTB. b) P values predicted by PaiNN based on the raw generated 3D structures versus DFT-optimized molecular structures.

Notes and references

[1] D. Weininger, *J. Chem. Inf. Comput. Sci.*, 1988, **28**, 31–36.

[2] G. Landrum, *RDKit: Open-source cheminformatics*, `http://www.rdkit.org/`, (accessed November 13, 2024).

[3] J. Westermayr, J. Gilkes, R. Barrett and R. J. Maurer, *Nat. Comput. Sci.*, 2023, **3**, 139–148.

[4] Z. Koczor-Benda, A. L. Boehmke, A. Xomalis, R. Arul, C. Readman, J. J. Baumberg and E. Rosta, *Phys. Rev. X*, 2021, **11**, 041035.

[5] Z. Koczor-Benda, P. Roelli, C. Galland and E. Rosta, *J. Phys. Chem. A*, 2022, **126**, 4657–4663.

[6] S. Genheden, A. Thakkar, V. Chadimová, J.-L. Reymond, O. Engkvist and E. Bjerrum, *J. Cheminform.*, 2020, **12**, 70.

[7] T. Sterling and J. J. Irwin, *J. Chem. Inf. Model.*, 2015, **55**, 2324–2337.

[8] A. Thakkar, T. Kogej, J.-L. Reymond, O. Engkvist and E. J. Bjerrum, *Chem. Sci.*, 2020, **11**, 154–168.

[9] D. Lowe, *Chemical reactions from US patents (1976-Sep2016)*, 2017, `https://doi.org/10.6084/m9.figshare.5104873.v1`, (accessed November 13, 2024).

[10] N. W. A. Gebauer, M. Gastegger and K. T. Schütt, in *Advances in Neural Information Processing Systems 32*, ed. H. Wallach, H. Larochelle, A. Beygelzimer, F. d'Alché Buc, E. Fox and R. Garnett, NeurIPS Proceedings, 2019, ch. Symmetry-adapted generation of 3d point sets for the targeted discovery of molecules.

[11] N. W. A. Gebauer, M. Gastegger, S. S. P. Hessmann, K.-R. Müller and K. T. Schütt, *Nat. Commun.*, 2022, **13**, 973.

[12] A. Stuke, C. Kunkel, D. Golze, M. Todorović, J. T. Margraf, K. Reuter, P. Rinke and H. Oberhofer, *Sci. Data*, 2020, **7**, 58.
